# Assessing the incidence of catastrophic health expenditure and impoverishment from out-of-pocket payments and their determinants in Bangladesh: evidence from the nationwide Household Income and Expenditure Survey 2016

**DOI:** 10.1093/inthealth/ihab015

**Published:** 2021-04-06

**Authors:** Sayem Ahmed, Mohammad Wahid Ahmed, Md Zahid Hasan, Gazi Golam Mehdi, Ziaul Islam, Clas Rehnberg, Louis W Niessen, Jahangir A M Khan

**Affiliations:** Liverpool School of Tropical Medicine, Liverpool, UK; Department of Learning, Informatics, Management and Ethics (LIME), Karolinska Institutet, Stockholm, Sweden; Oxford University Clinical Research Unit (OUCRU), Ho Chi Minh City 700000, Vietnam; Centre for Tropical Medicine and Global Health, Nuffield Department of Medicine, University of Oxford, Oxford OX3 7BN, UK; Health Economics and Financing Research, Health Systems and Population Studies Division, icddr, b, Dhaka, Bangladesh; Health Economics and Financing Research, Health Systems and Population Studies Division, icddr, b, Dhaka, Bangladesh; Health Economics and Financing Research, Health Systems and Population Studies Division, icddr, b, Dhaka, Bangladesh; Health Economics and Financing Research, Health Systems and Population Studies Division, icddr, b, Dhaka, Bangladesh; Department of Learning, Informatics, Management and Ethics (LIME), Karolinska Institutet, Stockholm, Sweden; Liverpool School of Tropical Medicine, Liverpool, UK; Department of International Health, Johns Hopkins School of Public Health, USA; Liverpool School of Tropical Medicine, Liverpool, UK; Department of Learning, Informatics, Management and Ethics (LIME), Karolinska Institutet, Stockholm, Sweden; School of Public Health and Community Medicine, University of Gothenburg, Gothenburg, Sweden

**Keywords:** Bangladesh, catastrophic health expenditure, healthcare financing, impoverishment, out-of-pocket payments

## Abstract

**Background:**

Out-of-pocket (OOP) payments for healthcare have been increasing steadily in Bangladesh, which deteriorates the financial risk protection of many households.

**Methods:**

We aimed to investigate the incidence of catastrophic health expenditure (CHE) and impoverishment from OOP payments and their determinants. We employed nationally representative Household Income and Expenditure Survey 2016 data with a sample of 46 076 households. A household that made OOP payments of >10% of its total or 40% of its non-food expenditure was considered to be facing CHE. We estimated the impoverishment using both national and international poverty lines. Multiple logistic models were employed to identify the determinants of CHE and impoverishment.

**Results:**

The incidence of CHE was estimated as 24.6% and 10.9% using 10% of the total and 40% of non-food expenditure as thresholds, respectively, and these were concentrated among the poor. About 4.5% of the population (8.61 million) fell into poverty during 2016. Utilization of private facilities, the presence of older people, chronic illness and geographical location were the main determinants of both CHE and impoverishment.

**Conclusion:**

The financial hardship due to OOP payments was high and it should be reduced by regulating the private health sector and covering the care of older people and chronic illness by prepayment-financing mechanisms.

## Introduction

In response to the United Nations Sustainable Development Goals (SDGs), the government of Bangladesh expressed a willingness to work to achieve Universal Health Coverage (UHC), which includes health service coverage (SDG 3.8.1) and financial risk protection (SDG 3.8.2).^1,2^ In recent years, Bangladesh has made remarkable progress in expanding coverage for essential public health interventions, such as immunization.^3^ However, Bangladesh has been facing difficulties in achieving the financial risk protection target because financing the country's healthcare relies heavily on out-of-pocket (OOP) payments.^4^

OOP payments are the primary source of healthcare financing in many low- and middle-income countries (LMICs), resulting in a financial burden on many households each year.^5,6^ Globally, around 150 million people experience catastrophic health expenditure (CHE) each year, and about 100 million individuals fall into poverty because of such payments.^7^ A majority (>90%) of impoverished people reside in LMICs.^5,7^ Therefore, the health systems of LMICs should aim to protect affected households from such payments to reduce the risk of impoverishment.^8^ SDG 3.8.2 measures ‘the proportion of the population with large household expenditure on health as a share of total household expenditure or income’.^9^ This deals with the affordability of accessing healthcare, which has important implications for reducing CHE from OOP payments.^8^

In recent years, a number of studies on OOP payments and their impact on CHE and impoverishment in different countries in Asia have been published.^10,11^ Evidence showed that the incidence of CHE is higher in low-income countries (3.1%) compared with middle- (1.8%) and high-income (0.6%) countries.^7^ Another study conducted in countries in Southeast Asia found that 242.7 million people faced CHE at a threshold level of 10% and that 58.2 million people faced impoverishment by falling below the poverty line of 1.90 international dollars per person per day.^12^ In 2008, Flores et al. estimated the incidence of CHE for inpatient care in India at 30%.^13^ Pandey et al. observed that the proportion of CHE increased by 2.24 times from 1995 to 2014 in India; this increase was higher in the poorest quintile than in the richest.^14^ In Myanmar, the incidence of CHE was estimated at 47% in 2015 using 40% of non-food expenditure as the threshold level.^15^ In 2017, the incidence of CHE was found to be 13% among industrial workers in Nepal.^15^ A study in Mongolia showed that despite the high coverage of social health insurance, approximately 5.5% of households incurred CHE, taking into consideration a threshold of 10% and that around 20 000 people were impoverished because of OOP payments.^16^ In 2007, a multi-country analysis showed that Bangladesh had the highest incidence of CHE (15.6%) among south Asian countries.^17^

In 2015, the government of Bangladesh spent little on health (US$8.5 per capita) while OOP expenditure remained high (US$37 per capita).^4^ It was further observed that OOP payments, as a percentage of total health expenditure, had been increasing steadily over the last few decades.^4^ Our previous research on the effects of OOP payments (60% of total health expenditure) in 2010 reported considerable financial risks for households.^4,18^ At the national level, 14.2% of households faced CHE and 3.5% people (5 million) fell into poverty because of OOP payments during that year. As a percentage of total health expenditure, OOP payments increased by 7% (from 60% to 67%) from 2010 to 2015.^4^ This inspired us to investigate the status of financial risk protection in households and society while OOP payments increased by employing the latest available national level data in Bangladesh. Estimating financial risk protection and comparing it with previous estimates (e.g. 2010) are essential for understanding the current situation and tracking the progress of the financial dimension of UHC in this country. Thus, we aimed to estimate the incidence of CHE and economic impoverishment from OOP payments for 2016 and identify their determinants. We compared the findings with countries that implemented social health insurance (e.g. Vietnam) over a similar period.

## Materials and Methods

### Data

Secondary data were obtained from the 2016 nationwide Household Income and Expenditure Survey (HIES) in Bangladesh.^19^ The HIES or household living standard survey is widely used globally, particularly in low-income developing countries, for assessing the level of poverty and living standards of people. In Bangladesh, the HIES is a repeated cross-sectional survey conducted by the Bangladesh Bureau of Statistics (BBS) over a 5-y period. This survey provides valuable data on matters such as household income, expenditure, consumption, savings, housing conditions, education, employment, health and sanitation, water supply and electricity. Data from the survey are also used to compile national accounts with regard to the household sector, and to analyze the poverty situation and other information regarding households. HIES 2016 employed a two-stage stratified random sampling technique. In the first stage, 2304 primary sampling units (PSUs) were selected using a probability proportional to size sampling technique from 64 districts of the country for national representation.^19^ In the second stage, 20 households were selected randomly from each PSU. Therefore, a total of 46 080 households (2304×20) were selected, of which 46 076 households responded to the survey.^19^ The household information on OOP payments for healthcare in the preceding 30 d and detailed household consumption expenditure from these surveys were utilized in the current study.

Because of the difference in the survey tools, OOP spending estimates may not be fully comparable between HIES 2010 and 2016. The BBS improved the module on OOP spending in HIES 2016 to collect accurate OOP spending data for various healthcare services (e.g. primary healthcare, outpatient department [OPD], inpatient department [IPD] and routine medication for chronic illness). It should be noted here that unlike HIES 2016, questions related to OOP spending were not asked separately for these items in HIES 2010.^19,20^ Therefore, caution should be taken when comparing the findings of the current study with the earlier one.

### CHE

OOP payments are defined as any payments made by households at the point of care (e.g. consultation fees, bed charges, diagnostic cost, medicine cost) and other related non-medical expenses (e.g. transportation, tips).^21,22^ Third-party payments (e.g. a payment made by micro health insurance) are not included in OOP healthcare payments. We estimated the incidence of CHE considering the fraction of a household’s OOP payments that exceeded certain thresholds of that household’s consumption expenditure.^23^ We used two different threshold levels to estimate the incidence of CHE: OOP payments exceeding (1) 10% of household total consumption expenditure and (2) 40% of household non-food expenditure. We considered both currency and in-kind payments on all goods and services, as well as the monetary value of the consumption of homemade products, as part of household consumption expenditure. Tobacco expenditure was included in the total expenditure estimation following the World Bank approach.

### Poverty lines and impoverishment

For poverty measurement, the national poverty line, provided by the BBS, and the international poverty line (IPL), were both employed in this study. The IPL was defined as 1.9 international dollars (purchasing power parity adjusted currency; 1 international dollar = 28.27 Bangladeshi Taka [BDT]^24^) spending per capita per day. The BBS applied the costs of basic need (CBN) approach to estimate the national poverty line.^20^ According to the CBN method, the poverty line represents the level of per capita expenditure at which household members are expected to meet their basic needs (consisting of food and non-food consumption items). The market prices of basic food necessities (11 food items, e.g. rice, wheat, pulses, milk, oil, meat, fish, potatoes, other vegetables, sugar and fruit comprising 2122 kcal per day per person) were captured for the food component of the poverty line. Furthermore, the non-food component of the poverty line was estimated using the median amount spent on non-food items by households as per capita food expenditure close to the food poverty line. Finally, the sum of the food and the non-food components constituted the poverty line. The impoverishment impact of OOP payments was estimated as the difference in poverty headcount estimated using ‘total household consumption expenditure’ and such expenditure without OOP payments for healthcare.^25^ Based on the estimated poverty line, a non-poor household was defined to be impoverished by OOP payments when it became poor after paying for healthcare services.^26^ The number of individuals pushed below the poverty line/impoverished from OOP payments was estimated by applying the proportion of impoverished to the total population in a particular year. Pen's parade graphs were drawn to present the impact of OOP payments on poverty. We used household consumption as multiples of the national poverty line and these are presented on the vertical axis of the graph. The downward vertical bar (or ‘paint drop’) for each household illustrates the extent to which the subtraction of OOP payments reduces consumption. A household is counted as poor based on reduced consumption because of OOP payments if the bar for that household crosses the poverty line.^23^ We adjusted CHE and impoverishment estimates for sample weights to more accurately reflect the entire population of the country. The sample weight representing the inverse of the probability of a household being selected for the sample was collected from the BBS. Multi-stage cluster designs of HIES 2016 were considered during sampling weight adjustment.^19^

### Asset quintiles

The living standard of each household was measured using the asset index. We used information on housing materials, access to basic facilities such as water and sanitation, durable goods and livestock to construct an index. During the analysis, the categorical asset variables were converted to dummy variables. We used principal component analysis (PCA) to determine the linear weighted combination of these asset variables.^27^ In PCA, the first principal component was used as an indicator of socioeconomic status.^25^ We divided the scores associated with the first principal into five quintiles as an indicator of the socioeconomic status of the household.^28,29^ In PCA, the effect of household size on asset score was adjusted using the number of household members as frequency weights.^30^

### Multiple regression analysis

We used two multiple logistic regression models, considering the CHE and impoverishment as separate dependent variables. We used socioeconomic characteristics (e.g. the head of the household’s gender and education level, household size, asset quintiles and geographical location), chronic illness and healthcare seeking from private facilities in these models as independent variables based on the literature.^13,18,31,32^ The first model was specified as

$$\begin{eqnarray*}
{\rm{logit}}({{\rm{Y}}_{\rm{i}}}) = {{\rm{\beta }}_0}{\rm{\,\,}} + {{\rm{\beta }}_1}{{\rm{X}}_{1{\rm{i}}}} + {{\rm{\beta }}_2}{{\rm{X}}_{2{\rm{i}}}} + \ldots\!\!\! &&+\, {{\rm{\varepsilon }}_{\rm{i}}} \ldots \ldots \ldots .{\rm{\,\,}}\left( {\rm{I}} \right);\\
&&{\rm{i}} = 1,2 \ldots {\rm{n}},\end{eqnarray*}$$

where Y_i_ denotes the dichotomous dependent variable with a value of 0 (i.e. the household did not face CHE) or 1 (i.e. the household faced CHE), β_0_ is a constant and X_1i_, X_2i_… denote the control variables. β_1_, β_2_, … denote adjacent coefficients to the corresponding independent variables and ${{\rm{\varepsilon }}_{\rm{i}}}$is the error term. We fitted four similar regression models using different dependent variables. In models 1 and 2, the dependent variables were the CHE status of a household estimated using two threshold levels (i.e. 10% of total expenditure and 40% of non-food expenditure, respectively). In models 3 and 4, we fitted impoverishment from OOP payments estimated using the national poverty line and international poverty line (1.9 international dollars per capita per day), respectively. ORs with 95% CIs were estimated to understand the association between dependent and independent variables in the multiple logistic regression models. Data were analyzed using the Stata, College Station, Texas, USA.

## Results

### Characteristics of households

The number of male-headed households was higher compared with those which were female-headed (Table 1). The majority (42.1%) of heads of households had no institutional education. A higher proportion of households had children; only 18.3% of households included older people. Most of the households (around 70%) were in rural areas and more than half of the households had 3–4 members. Almost half (48.8%) of the households had at least one member who sought healthcare for a chronic illness. More households had at least one member who utilized a public facility (12.8%), followed by utilization of a private facility (10.6%) or a non-governmental organization (NGO) facility (3.1%). About 8.1% of households had at least one member who utilized inpatient care in the last 1-y period.

**Table 1.** Characteristics of the sample

Variables	Percentage (95% CI), N=45 977
Gender of the head of the household	
Female	12.8 (12.4 to 13.1)
Male	87.2 (86.9 to 87.6)
Education level of the head of the household	
No institutional education	42.1 (41.6 to 42.5)
Up to primary	24.8 (24.4 to 25.2)
Secondary	24.8 (24.4 to 25.2)
Higher secondary	3.9 (3.8 to 4.1)
University	4.4 (4.2 to 4.6)
Having a child in the household	
No	28.0 (27.6 to 28.4)
Yes	72.0 (71.6 to 72.4)
Having an older person in the household	
No	81.7 (81.4 to 82.1)
Yes	18.3 (17.9 to 18.6)
Household size (equivalence scale)	
1–2	14.3 (14.0 to 14.6)
3–4	52.5 (52.0 to 52.9)
≥5	33.2 (32.8 to 33.7)
At least one member sought care for a chronic illness	
No	51.1 (50.7 to 51.6)
Yes	48.9 (48.4 to 49.3)
At least one household member utilized an inpatient service	
No	91.9 (91.7 to 92.2)
Yes	8.1 (7.8 to 8.3)
At least one household member utilized a public facility	
No	87.2 (86.9 to 87.5)
Yes	12.8 (12.5 to 13.1)
At least one household member utilized a private facility	
No	89.4 (89.1 to 89.7)
Yes	10.6 (10.3 to 10.9)
At least one household member utilized a non-governmental organization facility	
No	96.9 (96.8 to 97.1)
Yes	3.1 (2.9 to 3.2)
At least one household member utilized other providers	
No	61.2 (60.7 to 61.6)
Yes	38.8 (38.4 to 39.3)
Location	
Urban	30.3 (29.9 to 30.8)
Rural	69.7 (69.2 to 70.1)
Asset quintiles	
Poorest	20.0 (19.6 to 20.3)
Second	20.0 (19.6 to 20.4)
Third	20.0 (19.6 to 20.4)
Fourth	20.0 (19.6 to 20.4)
Richest	20.0 (19.6 to 20.4)

### OOP payments

Table 2 summarizes households’ OOP payments for 30 d. Around 75% of households made OOP payments in the last 30 d for OPD or in the last 1-y period for IPD services. The average household OOP payment on healthcare for 30 d was 1637 BDT (US$21) when the average was computed considering total population. When computed conditionally on who made any healthcare payment, the average OOP payment was 2174 BDT (US$28).

**Table 2.** Distribution of out-of-pocket (OOP) healthcare expenditure (at household level) over a 30-d period

Variables	Mean/percentage (95% CI)
Among the total population	
Average OOP payments in Bangladeshi Taka	1637.6 (1586.1 to 1689.2)
Average OOP payments in US$	20.9 (20.3 to 27.8)
OOP payments as a share of total expenditure	7.8% (7.7 to 8.0%)
OOP payments as a share of non-food expenditure	14.3% (14.1 to 14.4%)
Reported any health expenditure	75.3% (74.9 to 75.7%)
Conditional on making any healthcare payment	
Average OOP payments (in Bangladeshi Taka)	2174.0 (2106.6 to 2241.5)
Average OOP payments (in US$)	27.8 (26.9 to 28.6)
OOP payments as a share of total expenditure	10.4% (10.3 to 10.6%)
OOP payments as a share of non-food expenditure	18.9% (18.7 to 19.1%)

**Table 3.** The incidence of catastrophic health expenditure (CHE) using two threshold levels by demographic and socioeconomic characteristics

	CHE using 10% of total expenditure as the threshold level	CHE using 40% of non-food expenditure as the threshold level
Variables	% (95% CI)	% (95% CI)
Gender of the head of the household		
Female	26.0 (24.9 to 27.2)	11.7 (10.9 to 12.6)
Male	24.3 (23.9 to 24.8)	10.8 (10.5 to 11.1)
Education level of the head of the household		
No institutional education	24.7 (24.1 to 25.3)	12.1 (11.7 to 12.6)
Up to primary	25.5 (24.7 to 26.3)	11.1 (10.5 to 11.7)
Secondary	24.0 (23.2 to 24.8)	9.6 (9.1 to 10.2)
Higher secondary	22.4 (20.5 to 24.3)	8.2 (6.9 to 9.5)
University	22.7 (20.9 to 24.6)	7.3 (6.2 to 8.5)
Having a child in the household		
No	27.6 (26.8 to 28.4)	13.4 (12.8 to 14.0)
Yes	23.4 (22.9 to 23.8)	9.9 (9.6 to 10.2)
Having an older person in the household		
No	22.3 (21.9 to 22.7)	9.6 (9.3 to 9.9)
Yes	34.7 (33.7 to 35.7)	16.8 (16.0 to 17.6)
Household size (equivalence scale)		
1–2	28.4 (27.3 to 29.5)	15.3 (14.5 to 16.2)
3–4	22.6 (22.1 to 23.2)	9.6 (9.2 to 10.0)
≥5	26.0 (25.3 to 26.6)	11.0 (10.5 to 11.5)
At least one household member sought care for a chronic illness		
No	10.1 (9.7 to 10.5)	4.3 (4.1 to 4.6)
Yes	39.7 (39.0 to 40.3)	17.8 (17.3 to 18.3)
At least one household member utilized an inpatient service		
No	21.3 (20.9 to 21.7)	9.2 (9.0 to 9.5)
Yes	61.7 (60.1 to 63.3)	29.7 (28.2 to 31.1)
At least one household member utilized a public facility		
No	20.5 (20.1 to 20.9)	8.8 (8.5 to 9.1)
Yes	52.4 (51.2 to 53.7)	25.1 (24.0 to 26.2)
At least one household member utilized a private facility		
No	19.8 (19.4 to 20.2)	8.3 (8.1 to 8.6)
Yes	64.9 (63.6 to 66.2)	32.6 (31.3 to 33.9)
At least one household member utilized a non-governmental organization facility		
No	23.7 (23.3 to 24.1)	10.5 (10.2 to 10.8)
Yes	51.5 (48.9 to 54.1)	24.3 (22.1 to 26.6)
At least one household member utilized another provider (e.g. drug seller)/ self-treatment		
No	18.0 (17.5 to 18.4)	8.7 (8.4 to 9.1)
Yes	34.9 (34.2 to 35.6)	14.3 (13.8 to 14.8)
Location		
Urban	22.1 (21.4 to 22.8)	8.6 (8.1 to 9.0)
Rural	25.6 (25.1 to 26.1)	11.9 (11.6 to 12.3)
Asset quintiles		
Poorest	25.2 (24.3 to 26.1)	13.4 (12.7 to 14.1)
Second	25.5 (24.6 to 26.4)	12.2 (11.5 to 12.9)
Third	25.2 (24.3 to 26.1)	11.0 (10.4 to 11.7)
Fourth	24.8 (23.9 to 25.7)	10.1 (9.5 to 10.7)
Richest	22.0 (21.2 to 22.9)	6.3 (5.8 to 6.8)
Total	24.6 (24.2 to 24.9)	10.9 (10.6 to 11.2)

### CHE

The incidence of CHE is presented in Table 3 using two threshold levels by demographic and socioeconomic characteristics. Using 10% of the total health expenditure threshold level, the incidence of CHE was 24.6%, but using 40% of the non-food expenditure threshold level, it decreased to 10.9%. The incidence of CHE was higher among those households with at least one member who utilized a private facility compared with households with similar utilization of a public facility (using both definitions).

### Economic impoverishment

The economic impoverishment impact of OOP payments in 2016 is presented in Table 4. Around 4.50% of the population (or 8.61 million) was pushed below the national poverty line from OOP payments in 2016 (the relative difference in poverty due to OOP payments was 1.33%).

**Table 4.** Effect of out-of-pocket (OOP) spending on poverty headcounts

		Impoverishment due to OOP payment (Absolute difference)	
Poverty line	Measurement	Absolute difference	Relative difference
National poverty line	% population pushed below the national poverty line (95% CI)	4.50% (4.19 to 4.82%)	1.33% (1.24 to 1.41%)
	Number of individuals (in millions)	8.61	-
1.9 international dollar spending per capita per day as the poverty line	% population pushed below the international poverty line (95% CI)	2.02% (1.83 to 2.20%)	0.45% (0.42 to 0.49%)
	Number of individuals (in millions)	4.64	-

**Figure 1.**
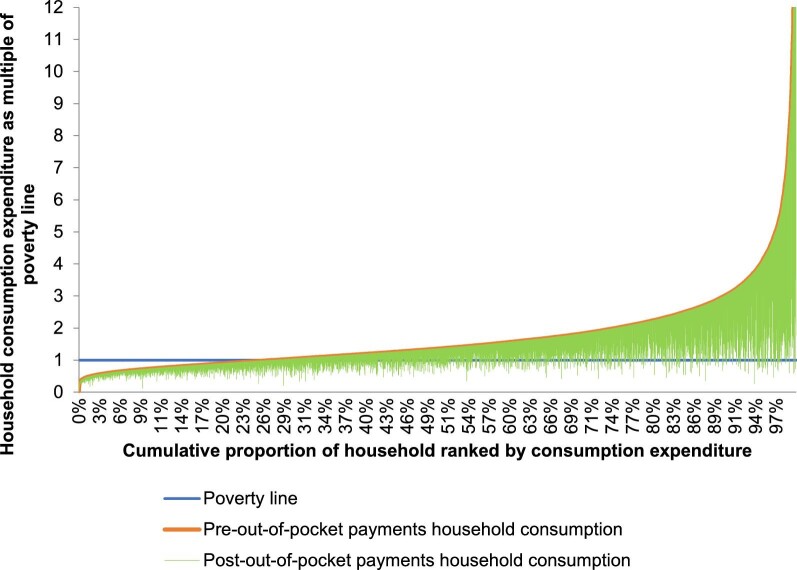
Poverty impact illustrated on a Pen's parade graph: before and after out-of-pocket (OOP) payments using the national poverty line.

The impact of OOP payments on poverty are presented in Figure 1. The graph shows that households in the middle and lower half of the distribution fell below the poverty line because of OOP payments they made in 2016. For those people who were already below the poverty line, their poverty status deteriorated further because of OOP payments.

### Multiple regression analysis

Table 5 shows the factors associated with the incidence of CHE using 10% of total household expenditure (model 1) and 40% of non-food expenditure threshold levels (model 2). The head of the household’s education level, having an older person in the household, household size, seeking healthcare for a chronic illness, utilization of private providers, geographical location and asset quintiles were significantly associated with the incidence of CHE. The ORs showed that a household in which the head of the household had an education up to university level was 0.770 (95% CI0.629 to 0.948) times less likely to experience CHE (using 40% of non-food expenditure as the threshold) than a household in which the head of the household had received no institutional education. Households with older members were more likely to face CHE (measured using both definitions) than households without older members.

**Table 5.** Factors associated with the incidence of catastrophic health expenditure (CHE)

		Model 1 (dependent=CHE using 10% of total expenditure)	Model 2 (dependent=CHE using 40% of non-food expenditure)
Variables	Description	OR (95% CI)	OR (95% CI)
Gender of the head of the household	Male (ref.=female)	0.95 (0.878 to 1.029)	1.017 (0.918 to 1.128)
Education level of the head of the household	Up to primary (ref.=no institutional education)	1.028 (0.964 to 1.097)	0.93 (0.856 to 1.010)
	Secondary (ref.=no institutional education)	1.011 (0.944 to 1.082)	0.912* (0.833 to 0.997)
	Higher secondary (ref.=no institutional education)	0.983 (0.852 to 1.135)	0.857 (0.701 to 1.047)
	University (ref.=no institutional education)	0.998 (0.867 to 1.150)	0.770* (0.626 to 0.948)
Having a child in the household	Yes (ref.=no)	0.897** (0.836 to 0.964)	0.900* (0.820 to 0.988)
Having an older person in the household	Yes (ref. =no)	1.317*** (1.236 to 1.403)	1.333*** (1.232 to 1.442)
Household size (equivalence	3–4 (ref.=1–2)	0.707*** (0.646 to 0.774)	0.621*** (0.554 to 0.695)
scale)	≥5 (ref.=1–2)	0.586*** (0.528 to 0.650)	0.535*** (0.468 to 0.610)
At least one member sought care for a chronic illness	Yes (ref.=no)	4.692*** (4.434 to 4.966)	3.703*** (3.419 to 4.010)
At least one household member utilized an inpatient service	Yes (ref.=no)	1.079 (0.978 to 1.190)	0.96 (0.865 to 1.067)
At least one household member utilized a public facility	Yes (ref.=no)	4.117*** (3.807 to 4.452)	3.260*** (2.980 to 3.567)
At least one household member utilized a private facility	Yes (ref.=no)	9.880*** (9.010 to 10.83)	6.852*** (6.216 to 7.552)
At least one household member utilized a non-governmental organization facility	Yes (ref.=no)	0.131*** (0.111 to 0.153)	0.193*** (0.163 to 0.229)
At least one household member utilized another provider	Yes (ref.=no)	2.717*** (2.578 to 2.863)	1.761*** (1.646 to 1.884)
Location	Rural (ref.=urban)	1.046 (0.982 to 1.113)	1.067 (0.981 to 1.159)
Division	Chittagong (ref.=Barisal)	0.643*** (0.584 to 0.707)	0.530*** (0.471 to 0.595)
	Dhaka (ref.=Barisal)	0.467*** (0.425 to 0.513)	0.490*** (0.436 to 0.550)
	Khulna (ref.=Barisal)	0.435*** (0.395 to 0.479)	0.415*** (0.368 to 0.468)
	Rangpur (ref.=Barisal)	0.465*** (0.408 to 0.530)	0.517*** (0.440 to 0.607)
	Rajshahi (ref.=Barisal)	0.486*** (0.444 to 0.531)	0.444*** (0.399 to 0.495)
	Sylhet (ref.=Barisal)	0.410*** (0.362 to 0.465)	0.310*** (0.261 to 0.368)
Asset quintiles	Second (ref.=poorest)	0.974 (0.901 to 1.054)	0.854** (0.776 to 0.941)
	Third (ref.=poorest)	0.889** (0.820 to 0.963)	0.720*** (0.651 to 0.796)
	Fourth (ref.=poorest)	0.785*** (0.722 to 0.854)	0.595*** (0.535 to 0.663)
	Richest (ref.=poorest)	0.630*** (0.571 to 0.694)	0.328*** (0.287 to 0.376)
Constant		0.163*** (0.142 to 0.188)	0.108*** (0.091 to 0.129)
N		45 289	45 289
Log-likelihood (LR)		−19 057	−12 406
LR χ^2^		12 371	5 797.1
Degrees of freedom		26	26
p>χ^2^		<0.000	<0.000
Pseudo R^2^		0.245	0.189

*p<0.05, **p<0.01, ***p<0.001.

Larger households were less likely to observe an incidence of CHE compared with smaller ones, in both models. Households in which at least one member had a chronic illness were more likely to experience CHE than households without chronic illness (OR=5.271; 95% CI 4.992 to 5.564 in model 1 and OR=4.215, 95% CI 3.900 to 4.557 in model 2). Households that had utilized a private facility in the last 30 d had a 9.880 (95% CI 9.010 to 10.830) times higher chance of facing CHE using the 10% threshold level (model 1). A similar finding was observed (OR=6.852; 95% CI 6.216 to 7.552) using the 40% threshold level (model 2). The risk of facing CHE also varied significantly with the region of residence. Residents from the Barisal division had a significantly higher risk of facing CHE than all the other divisions. Households belonging to the highest asset quintile had significantly lower chances of facing CHE compared with the lowest quintile in both models.

Table 6 presents the determinants of impoverishment from OOP payments using the national poverty line (model 3) and 1.9 international dollars per person per day as the poverty line (model 4). Determinants of economic impoverishment were identified through these models: the education level of the head of the household, having children and older people in the household, larger household size, chronic illness, utilization of a private provider, rural residence and geographical location. Households with educated heads were less likely to fall below the poverty line because of OOP payments. Considering the national poverty line (model 3), if a household head had a university-level education, then that household was 0.236 times less likely (95% CI 0.166 to 0.337) to face impoverishment compared with a household in which its head had not received any institutional education. Similarly, when considering the international poverty line, if a household head had a university-level education then that household was 0.114 times less likely (95% CI 0.056 to 0.230) to face impoverishment compared with households where the head of household had received no institutional education. Households with at least one member with a chronic illness had a 2.275 (95% CI 2.073 to 2.498) times higher risk of impoverishment compared with those households without any members experiencing chronic illness. Utilization of healthcare from a private provider also increased the risk of impoverishment for a household by 2.221 (95% CI 1.958 to 2.543) times when considering the national poverty line, and by 1.803 (95% CI 1.492 to 2.179) times when considering the international poverty line, compared with those households without such utilization. Rural households were 1.469 (95% CI 1.328 to 1.626) times more likely to be impoverished compared with urban households (model 3). The households in the Barisal division had a significantly higher risk of falling into impoverishment than the households in other divisions.

**Table 6.** Factors associated with the impoverishment from out-of-pocket (OOP) healthcare spending

		Model 3 (dependent=impoverishment due to OOP payment using the national poverty line)	Model 4 (dependent=impoverishment due to OOP payment using 1.9 international dollar as the poverty line)
Variables	Description	OR (95% CI)	OR (95% CI)
Gender of the head of the household	Male (ref.=female)	1.034 (0.901 to 1.186)	0.885 (0.733 to 1.070)
Education level of the head of the household	Up to primary (ref.=no institutional education)	0.891* (0.809 to 0.981)	0.702*** (0.616 to 0.801)
	Secondary (ref.=no institutional education)	0.608*** (0.544 to 0.679)	0.426*** (0.363 to 0.501)
	Higher secondary (ref.=no institutional education)	0.281*** (0.200 to 0.395)	0.148*** (0.0787 to 0.277)
	University (ref.=no institutional education)	0.236*** (0.166 to 0.337)	0.114*** (0.056 to 0.230)
Having a child in the household	Yes (ref.=no)	1.481*** (1.307 to 1.678)	2.095*** (1.731 to 2.537)
Having an older person in the household	Yes (ref.=no)	1.250*** (1.132 to 1.382)	1.184* (1.032 to 1.359)
Household size (equivalence	3–4 (ref.=1–2)	0.970 (0.822 to 1.144)	0.997 (0.774 to 1.283)
scale)	≥5 (ref.=1–2)	1.058 (0.883 to 1.268)	1.314* (1.004 to 1.721)
At least one member sought care for a chronic illness	Yes (ref.=no)	2.275*** (2.073 to 2.498)	2.043*** (1.803 to 2.315)
At least one household member utilized an inpatient service	Yes (ref.=no)	0.963 (0.838 to 1.107)	0.811* (0.665 to 0.989)
At least one household member utilized a public facility	Yes (ref.=no)	2.214*** (1.981 to 2.475)	2.057*** (1.766 to 2.396)
At least one household member utilized a private facility	Yes (ref.=no)	2.231*** (1.958 to 2.543)	1.803*** (1.492 to 2.179)
At least one household member utilized a non-governmental organization facility	Yes (ref.=no)	0.506*** (0.409 to 0.625)	0.696* (0.522 to 0.927)
At least one household member utilized other providers	Yes (ref.=no)	1.747*** (1.608 to 1.897)	1.919*** (1.713 to 2.151)
Location	Rural (ref.=urban)	1.469*** (1.328 to 1.626)	1.871*** (1.607 to 2.177)
Division	Chittagong (ref.=Barisal)	0.388*** (0.331 to 0.453)	0.370*** (0.297 to 0.462)
	Dhaka (ref.=Barisal)	0.430*** (0.369 to 0.501)	0.369*** (0.295 to 0.462)
	Khulna (ref.=Barisal)	0.710*** (0.616 to 0.818)	0.852 (0.702 to 1.034)
	Rangpur (ref.=Barisal)	0.677*** (0.555 to 0.824)	0.957 (0.748 to 1.225)
	Rajshahi (ref.=Barisal)	0.749*** (0.658 to 0.852)	0.926 (0.778 to 1.103)
	Sylhet (ref.=Barisal)	0.503*** (0.414 to 0.610)	0.339*** (0.251 to 0.457)
Constant		0.023*** (0.018 to 0.028)	0.008*** (0.006 to 0.011)
N		45 968	45 968
Log-likelihood (LR)		−9156	−5502
LR χ^2^		1900	1308
Degrees of freedom		22	22
p>χ^2^		<0.000	<0.000
Pseudo R^2^		0.094	0.106

*p<0.05, **p<0.01, ***p<0.001.

## Discussion

The findings in this study indicate that attempts to achieve the financial risk protection target (SDG 3.8.2) deteriorated in 2016, which might have been influenced by increasing reliance on OOP payments for healthcare in Bangladesh. The incidence of CHE was estimated to be 24.6% (using 10% of total household expenditure as the threshold level) and 10.9% (using 40% of non-subsistence expenditure as the threshold level) in the current study. In 2017, Khan et al. estimated that the incidence of CHE in 2010 was 14.2% and 9.7% (using the respective definitions for CHE).^18^ We observed a 10.4% increase in the incidence of CHE (using the 10% threshold) from 2010 to 2016. The increase in the incidence of CHE can be explained by the increased reliance on OOP spending on healthcare financing in Bangladesh. Bangladesh National Health Accounts for 2015 reported that the share of OOP spending in total health expenditure increased from 60% to 67% from 2010 to 2015, while the share of government spending in total health expenditure reduced from 26% to 23% during the same period.^4^

We observed that having an older person in the household was significantly associated with a higher incidence of CHE. This may be because older people are more vulnerable to any illness, including chronic conditions and geriatric health problems, resulting in higher OOP spending than by the adult members of the household.^33–37^ A large household size was associated with a lower incidence of CHE because that household might have more than one earning member and consequently a higher total household income and expenditure. Those households with at least one member who utilized services for a chronic disease were 4.7 times more likely (95% CI 4.4 to 5.0) to face CHE than those households without any such member. The routine medication and complicated long-term hospitalization because of chronic illness might have incurred high OOP spending and CHE in those households.^38^ The utilization of private facilities was positively associated with CHE because of the high price of treatment in those facilities.^39^ Public facility utilization was also significantly associated with the incidence of CHE because a household might have made OOP payments to purchase certain medical care items from the private market during their stay in public facilities, mainly due to stock out of the required medicine and the unavailability of other services (e.g. diagnosis).^40^ The utilization of NGO facilities was associated with a lower incidence of CHE (OR=0.131, 95% CI 0.111 to 0.153). In many cases, the services in NGO facilities were subsidized through donor support, which might have reduced the OOP spending of households when utilizing this type of facility.^41^ Some NGOs (e.g. BRAC, the Sajeda Foundation) offer micro-insurance along with their micro-credit program in Bangladesh, which results in limited financial risk protection for the enrolled households.^42^ However, these facilities cover a smaller proportion of patients (0.61%) compared with private (69.6%) and public (27.5%) facilities. Also, these facilities might not be sustainable due to their reliance on donor funding.^41,43^

Our analysis of CHE incidences across socioeconomic quintiles found the highest proportion of CHE among the poorest group of households. The poorest households had lower spending capacity (lowest expenditure level) and any OOP spending constituted a large proportion of their total expenditure. Therefore, they were more prone to face CHE in the absence of any safety net program to cover healthcare expenses.^44,45^ The lower incidence of CHE among those households with inpatient service utilization should be interpreted with caution. The OOP spending on such treatment was higher than other types of care and therefore the incidence of CHE should be high in this group. However, HIES 2016 reported annual OOP spending information for inpatient care, which was underestimated when converted to monthly spending for comparisons with total monthly household expenditure so as to estimate the incidence of CHE.^46^

The findings of the current study are in line with previous studies of CHE and economic impoverishment in Bangladesh.^17,18,38,47^ Van Doorslaer et al., using data from HIES 2000, found that 15.6% and 7.1% of households in Bangladesh faced CHE considering 10% of total expenditure and 40% of non-food expenditure as thresholds, respectively.^17^ Unlike the current study, the authors observed a higher incidence of CHE among richer households. The reason for this difference may be due to the use of expenditure quintiles as the measure of socioeconomic status, whereas we used asset quintiles. Rahman et al found that 9% of households in a metropolitan city of Bangladesh faced CHE due to health spending and that such a catastrophe was four times higher in the poorest households than in the richest ones.^38^ India, a neighboring country, also experienced a higher risk of CHE among households with older people.^14^ In some Asian countries, households with older people and one member who had a chronic disease were at a higher risk of experiencing CHE.^48,49^ It was observed that even in the 15 wealthiest countries in Europe, those households with older people with chronic illnesses incurred CHE more frequently.^50^ Smaller households with older people faced a higher risk of CHE in urban Nigeria.^51^ On the other hand, the incidence of CHE was higher in rural compared with urban areas.^52,53^

The measurement of poverty was applied differently in different studies.^18,21,47^ We used both ‘CBN’ and ‘international dollar (1.9 per capita per day)’-based poverty lines for measuring the poverty outcomes in the current study, although we put more emphasis on the former because it considers the local price level of household consumption. The same poverty line was also used by the BBS for estimating the poverty headcount in Bangladesh.^20^ Hamid et al., using data from low-income rural people, found that 3.4% of people fell into poverty due to OOP spending on healthcare; their study identified non-communicable diseases (NCDs), hospitalization and catastrophic illness as the major reasons behind economic impoverishment.^47^ Khan et al. estimated a 3.5% difference in the poverty headcount (5.1 million individuals) due to OOP payments in 2010 using the CBN approach.^18^ Compared with the findings of Khan et al., we found a 1% increase in impoverishment due to OOP payments in 2016. Remarkably, from 2010 to 2016, the national poverty of Bangladesh decreased from 31.5% to 24.3% (a 7.2% decrease).^20,54^ This tends to suggest that the financial risk protection system in the country has not improved over the years and that people are inequitably becoming poor due to healthcare payments, despite the national progress in poverty alleviation. It should be noted that this estimate considered only the population above the poverty line that fell below the line due to OOP payments. Due to their incapacity to pay, the population below the poverty line may not utilize healthcare, and those who utilize healthcare may have worsened their poverty status, as reflected in Figure 1. The education level of the head of the household, household size, the number of dependents in the households and illness are widely recognized as the determinants of overall poverty in Bangladesh.^55,56^ We found similar determinants of impoverishment for OOP payments in the current study. Khan et al. and Mirelman et al. found an association between chronic disease and overall poverty in rural Bangladesh.^57–59^ The current study reports a similar association between chronic illness and OOP-related poverty. Besides current poverty-alleviation programs, healthcare financing in Bangladesh should reduce the reliance on OOP healthcare payments in order to reduce CHE and, consequently, poverty.

We used the latest survey data from HIES 2016 to estimate CHE incidence and economic impoverishment. In this round of the survey, the Bangladesh Statistics Bureau included the health expenditure module, which provides a unique opportunity to estimate these indicators of financial risk protection more accurately. The main limitation of the current study is that it is based on cross-sectional data. Ideally, longitudinal data should be used to assess the causal effect of OOP spending on the economic impoverishment of households.^60^ Using cross-sectional data, only point estimations could be performed and, consequently, we could not determine what proportion of households faced persistent impoverishment. It is possible that some households were only in a CHE condition or poverty for a short time.

### Conclusions

The dependence on OOP payments as a healthcare-financing mechanism in Bangladesh exposes households to financial risk, especially the poorest households. The current study found key drivers of CHE and impoverishment, which draws attention to the need to address the various social determinants of health so as to improve financial risk protection. This requires adopting a multi-sectoral approach that would effectively engage and regulate the private sector in service delivery, as well as addressing the burden of NCDs and other diseases through a primary healthcare approach. Policies and strategies such as the Healthcare Financing Strategy and the National Social Security Strategy adopted by the government of Bangladesh need to be urgently translated into action. However, these strategies are adversely affected by implementation shortfalls.^61,62^ The government, development partners and program implementers should set priorities for funding and implementing prepayment schemes, like social health insurance and micro health insurance, to mitigate the negative effects of OOP payments. Also, the government of Bangladesh should consider increasing its contribution to the health sector through tax funding to reduce OOP payment dependency.

## Data Availability

Data underlying this article were obtained from the Bangladesh Bureau of Statistics. Data can be shared upon request to the corresponding author with the permission of the Bangladesh Bureau of Statistics.

## References

[1] UN . Transforming Our World: The 2030 Agenda for Sustainable Development. New York; 2015.

[2] MoHFW . Expanding Social Protection for Health: Towards Universal Coverage, Health Care Financing Strategy 2012–2032. Dhaka; 2012.

[3] Balabanova D, Mills A, Conteh L et al. Good health at low cost 25 years on: Lessons for the future of health systems strengthening. Lancet. 2013;381(9883):2118–33. 23574803 10.1016/S0140-6736(12)62000-5

[4] MoHFW . Bangladesh National Health Accounts (BNHA) 1997–2015. Dhaka; 2018. Available at http://www.searo.who.int/bangladesh/bnha/en/ [accessed October 25, 2018].

[5] Xu K, Evans DB, Kawabata K et al. Household catastrophic health expenditure: a multicountry analysis. Lancet. 2003;362(9378):111–7. 12867110 10.1016/S0140-6736(03)13861-5

[6] Mills A. Health care systems in low- and middle-income countries. N Engl J Med. 2014;370(6):552–7. 24499213 10.1056/NEJMra1110897

[7] Xu K, Evans DB, Carrin G et al. Protecting households from catastrophic health spending. Health Aff. 2007;26(4):972–83. 10.1377/hlthaff.26.4.972 17630440

[8] WHO . The World Health Report: Health Systems Financing: The Path to Universal Coverage. Geneva: World Health Organisation; 2010. 10.2471/BLT.10.078741 PMC2878164 20539847

[9] WHO . Monitoring Sustainable Development Goals –Indicator 3.8.2. Available at https://www.who.int/health_financing/topics/financial-protection/monitoring-sdg/en/ [accessed January 29, 2021].

[10] Tomini SM, Packard TG, Tomini F. Catastrophic and impoverishing effects of out-of-pocket payments for health care in Albania: evidence from Albania Living Standards Measurement Surveys 2002, 2005 and 2008. Health Policy Plan. 2012;28(4):419–28. 22907092 10.1093/heapol/czs073

[11] Amaya Lara JL, Ruiz Gómez F. Determining factors of catastrophic health spending in Bogota, Colombia. Int J Health Care Finance Econ. 2011;11(2):83–100. 21359837 10.1007/s10754-011-9089-3

[12] Wang H, Torres LV, Travis P. Financial protection analysis in eight countries in the WHO South-East Asia Region. Bull World Health Organ. 2018;96(9):610–20. 30262942 10.2471/BLT.18.209858 PMC6154066

[13] Flores G, Krishnakumar J, O'Donnell O et al. Coping with health-care costs: implications for the measurement of catastrophic expenditures and poverty. Heal Econ. 2008;17(12):1393–1412. 10.1002/hec.1338 18246595

[14] Pandey A, Ploubidis GB, Clarke L et al. Trends in catastrophic health expenditure in india: 1993 to 2014. Bull World Health Organ. 2018;96(1):18–28. 29403097 10.2471/BLT.17.191759 PMC5791868

[15] Myint C-Y, Pavlova M, Groot W. Catastrophic health care expenditure in Myanmar: policy implications in leading progress towards universal health coverage. Int J Equity Health. 2019;18(1):1–13. 31362749 10.1186/s12939-019-1018-y PMC6664746

[16] Dorjdagva J, Batbaatar E, Svensson M et al. Catastrophic health expenditure and impoverishment in Mongolia. Int J Equity Health. 2016;15(1):105. 27401464 10.1186/s12939-016-0395-8 PMC4939814

[17] Van Doorslaer E, O'Donnell O, Rannan-Eliya RP et al. Catastrophic payments for health care in Asia. Heal Econ. 2007;16(11):1159–84. 10.1002/hec.1209 17311356

[18] Khan J, Ahmed S, Evans TG. Catastrophic healthcare expenditure and poverty related to out-of-pocket payments for healthcare in Bangladesh-an estimation of financial risk protection of universal health coverage. Health Policy Plan. 2017;32(8):1102–10. 28575415 10.1093/heapol/czx048

[19] BBS . Household Income & Expenditure Survey 2016. Dhaka; 2017.

[20] BBS . Household Income and Expenditure Survey 2010. Dhaka; 2011.

[21] van Doorslaer E, O'Donnell O, Rannan-Eliya RP et al. Effect of payments for health care on poverty estimates in 11 countries in Asia: an analysis of household survey data. Lancet. 2006;368(9544):1357–64. 17046468 10.1016/S0140-6736(06)69560-3

[22] WHO & WB . Tracking Universal Health Coverage: 2017 Global Monitoring Report. Geneva; 2017.

[23] Wagstaff A, van Doorslaer E. Catastrophe and impoverishment in paying for health care: with applications to Vietnam 1993–1998. Health Econ. 2003;12(11):921–34. 14601155 10.1002/hec.776

[24] WDI . World Development Indicators. The World Bank. Washington DC; 2016.

[25] O'Donnell O, van Doorslaer E, Wagstaff A et al. Analyzing Health Equity Using Household Survey Data: A Guide to Techniques and Their Implementation. Washington DC: The World Bank; 2008.

[26] Xu K. Distribution of Health Payments and Catastrophic Expenditures Methodology. Geneva; 2005.

[27] Vyas S, Kumaranayake L. Constructing socio-economic status indices: how to use principal components analysis. Health Policy Plan. 2006;21(6):459–68. 17030551 10.1093/heapol/czl029

[28] Filmer D, Pritchett LH. Estimating wealth effects without expenditure data–or tears: an application to educational enrollments in states of India. Demography. 2001;38(1):115–32. 11227840 10.1353/dem.2001.0003

[29] Gwatkin D, Wagstaff A, Yazbeck A. Reaching the Poor with Health, Nutrition, and Population Services: What Works, What Doesn't, and Why. Washington: World Bank; 2005.

[30] Deaton A, Paxson C. Economies of scale, household size, and the demand for food. J Pol Econ. 1998;106(5):897–930.

[31] Ahmed S, Szabo S, Nilsen K. Catastrophic healthcare expenditure and impoverishment in tropical deltas: evidence from the Mekong Delta region. Int J Equity Health. 2018;17(1):53. 29703209 10.1186/s12939-018-0757-5 PMC5924496

[32] Van Minh H, Kim Phuong NT, Saksena P et al. Financial burden of household out-of pocket health expenditure in Viet Nam: findings from the National Living Standard Survey 2002–2010. Soc Sci Med. 2013;96(2013):258–63. 23246399 10.1016/j.socscimed.2012.11.028

[33] Chowdhury MAH, Haque MR. Geriatric health problems of the elderly Garo people in Madhupur Upazila of Tangail District. Soc Sci Rev. 2013;30(1):77–86.

[34] Hypertension Study Group . Prevalence, awareness, treatment and control of hypertension among the elderly in Bangladesh and India: a multicentre study. Bull World Health Organ. 2001;79(6):490–500. 11436469 PMC2566443

[35] Wang Y, Jiang Y, Li Y et al. Health insurance utilization and its impact: observations from the middle-aged and elderly in china. PLoS One. 2013;8(12):e80978. 24324654 10.1371/journal.pone.0080978 PMC3855696

[36] Khanam MA, Streatfield PK, Kabir ZN et al. Prevalence and Patterns of Multimorbidity among Elderly People in Rural Bangladesh : a cross-sectional Study. J Health Popul Nutr. 2015;29(4):406–14. 10.3329/jhpn.v29i4.8458 PMC3190372 21957680

[37] Mayhew L. Health and Elderly Care Expenditure in an Aging World. International Institute for Applied Systems Analysis. Laxenburg; 2000.

[38] Rahman MM, Gilmour S, Saito E et al. Health-related financial catastrophe, inequality and chronic illness in Bangladesh. PLoS One. 2013;8(2):e56873. 23451102 10.1371/journal.pone.0056873 PMC3581555

[39] Pavel MS, Chakrabarty S, Gow J. Cost of illness for outpatients attending public and private hospitals in Bangladesh. Int J Equity Health. 2016;15(1):167. 27724955 10.1186/s12939-016-0458-x PMC5057498

[40] Mannan M. Access to Public Health Facilities in Bangladesh: a study on facility utilisation and burden of treatment. Bangladesh Dev Stud. 2013;36(4):25–80.

[41] Sarwar MR. Bangladesh Health Service Delivery: innovative NGO and private sector partnerships. IDS Bull. 2015;46(3):17–28.

[42] Ahsan SM, Hamid SA, Barua S et al. Working Paper No. 17 Micro Health Insurance in Bangladesh : Innovations in Design, Delivery and Distribution Channels. Dhaka; 2013.

[43] BBS . Health and Morbidity Status Survey. Dhaka; 2014.

[44] Haider MZ, Mahamud A. Beneficiary selection and allowance utilization of social safety net programme in Bangladesh. J Hum Rights Soc Work. 2017;2(1-2):45–51.

[45] Rahaman HZ, Choudhury LA. Social Safety Nets in Bangladesh Volume 2 Ground Realities and Policy Challenges Process Coverage Outcomes Priorities. Vol 02. Dhaka; 2012.

[46] Chuma J, Maina T. Catastrophic health care spending and impoverishment in Kenya. BMC Health Serv Res. 2012;12(1):413. 23170770 10.1186/1472-6963-12-413 PMC3561146

[47] Hamid SA, Ahsan SM, Begum A. Disease-specific impoverishment impact of out-of-pocket payments for health care: evidence from rural Bangladesh. Appl Health Econ Health Policy. 2014;12(4):421–33. 24854546 10.1007/s40258-014-0100-2

[48] Ghimire M, Ayer R, Kondo M. Cumulative incidence, distribution, and determinants of catastrophic health expenditure in Nepal: results from the living standards survey. Int J Equity Health. 2018;17(1):23. 29444686 10.1186/s12939-018-0736-x PMC5813388

[49] Choi JW, Choi JW, Kim JH et al. Association between chronic disease and catastrophic health expenditure in Korea. BMC Health Serv Res. 2015;15(1):1–8. 25608983 10.1186/s12913-014-0675-1 PMC4307618

[50] Arsenijevic J, Pavlova M, Rechel B et al. Catastrophic health care expenditure among older people with chronic diseases in 15 European countries. PLoS One. 2016;11(7):1–18. 10.1371/journal.pone.0157765 PMC4933384 27379926

[51] Adisa O. Investigating determinants of catastrophic health spending among poorly insured elderly households in urban Nigeria.[Erratum appears in Int J Equity Health. 2015;14:104. J Equity Heal. 2015;14:79. 26503100 10.1186/s12939-015-0241-4 PMC4620639

[52] Somkotra T, Lagrada LP. Payments for health care and its effect on catastrophe and impoverishment: Experience from the transition to Universal Coverage in Thailand. Soc Sci Med. 2008;67(12):2027–35. 18952336 10.1016/j.socscimed.2008.09.047

[53] Abu-Zaineh M, Romdhane H Ben, Ventelou B et al. Appraising financial protection in health: The case of Tunisia. Int J Health Care Finance Econ. 2013;13(1):73–93. 23381233 10.1007/s10754-013-9123-8

[54] BBS . Preliminary report on Households Income and Expenditure Survey 2016. 2017:0–2. Available at https://catalog.ihsn.org/index.php/catalog/7399/related-materials [accessed 29 January 2021].

[55] Newhouse D, Suarez-Becerra P, Evans MC. New Estimates of Extreme Poverty for Children. Washington D.C.; 2016.

[56] Imam MF, Islam MA, Hossain M. Factors affecting poverty in rural Bangladesh: An analysis using multilevel modelling. J Bangladesh Agric Univ. 2018;16(1):123–30.

[57] Khan JAM, Trujillo AJ, Ahmed S et al. Distribution of chronic disease mortality and deterioration in household socioeconomic status in rural Bangladesh: an analysis over a 24-year period. Int J Epidemiol. 2015;44(6):1917–26. 26467760 10.1093/ije/dyv197 PMC5156339

[58] Mirelman AJ, Rose S, Khan JA et al. The relationship between non-communicable disease occurrence and poverty—evidence from demographic surveillance in Matlab, Bangladesh. Health Policy Plan. 2016;31(6):785–92. 26843515 10.1093/heapol/czv134

[59] Mirelman AJ, Trujillo AJ, Niessen LW et al. Household coping strategies after an adult noncommunicable disease death in Bangladesh. Int J Health Plann Manage. 2019 (January);34(1):e203–e218. 30187582 10.1002/hpm.2637

[60] Sauerborn R, Adams A, Hien M. Household strategies to cope with the economic costs of illness. Soc Sci Med. 1996;43(3):291–301. 8844932 10.1016/0277-9536(95)00375-4

[61] Ahmed S, Hasan MZ, Ahmed MW et al. Evaluating the implementation related challenges of Shasthyo Suroksha Karmasuchi (health protection scheme) of the government of Bangladesh: a study protocol. BMC Health Serv Res. 2018;18(1):552. 30012139 10.1186/s12913-018-3337-x PMC6048757

[62] Joarder T, Chaudhury TZ, Mannan I. Universal health coverage in Bangladesh: activities, challenges, and suggestions. Adv Public Heal. 2019;2019:1–12. 10.1155/2019/4954095 PMC7691757 33281233

